# Some Medico-Legal Reminiscences

**Published:** 1907-11-30

**Authors:** Arthur P. Luff

**Affiliations:** Physician to St. Mary's Hospital.


### Hospital Clinics.

### SOME MEDICO-LEGAL REMINISCENCES.

By ARTHUR P. LUFF, M.D., B.Sc., F.R.C.P. (Lond.), Physician to St. Mary's Hospital.

A Lecture delivered at the Polyclinic and Medical Graduates' College.

When, three years ago, I resigned my post in connection with the Home Office, after an experience of seventeen years of medico-legal work, it was suggested to me that some of my reminiscences might be of interest. I have been connected with many cases of an extremely sensational character, which unfortunately appeal very much to the public; but I am not going to deal with sensation in the brief discourse this afternoon. In the course of these cases I have seen a large number of medical men in the witness box, and I have been struck with the imperfect manner in which some of them give evidence. A fair proportion, in giving evidence, do not compare favourably with other educated witnesses; this is due to two reasons. First, they are too verbose. A good witness should be as brief as possible. The lengthy and verbose answer is likely to lead to severe cross-examination. Secondly, they are too prone to use technical terms. The answer should be such as is perfectly intelligible to the meanest juryman present, for in criminal trials the jury is not always selected from a very intelligent class, and the answer of a medical witness should be as free as possible from technical details. I remember perfectly well, in a trial for poisoning before Baron Huddlestone—the last of the Barons on the judicial bench of this country—a medical witness was asked to state what he had given his patient. In a very halting and hesitating way, he stated that he had given " . . . a nervine sedative, and a . . . gastric carminative." Baron Huddlestone turned round upon him and asked, " Will you tell us what you did give?" It turned out that he had administered a little bismuth and cinnamon water. A medical witness in another case hesitated still more, and the judge said to him, " The law of this country is that no individual is bound to incriminate himself; if you consider that the antidote killed the person, don't say what you gave."

The cases that I have selected are six in number, and I have taken them in the chronological order in which they occurred. The first one raised a point of importance in connection with dying declarations. I shall have to detail a little the facts of the case, so that you can understand the way this point arose. It was in connection with a member of our profession, who was tried for criminal abortion, and was acquitted of the charge; it is therefore undesirable to mention his name. A dying declaration is the one instance in which hearsay evidence is allowed in a court of law as equivalent to evidence ordinarily given on oath. The statement of the dying person can be taken in a court of law as evidence after the death of the person, and is equivalent in its value to evidence given in the witness box, provided it is correctly taken down. Everything depends on that. The person making the declaration must be absolutely certain that death is about to occur, and that recovery is absolutely impossible. Unless these points are expressed in a dying declaration, it is not evidence in a court of law. The law assumes that a statement then made will be a true one, and that a dying person will not falsely incriminate another individual.

In this case, the question that arose was whether a dying declaration can be invalidated by a subsequent statement of the person who made it. The case was that of an attractive girl, approximately about twenty-four years of age, who apparently had lived a moral life prior to her meeting with the defendant. Her statement was that, on one occasion, outside Victoria Station, she had met him quite by chance, that an acquaintance had sprung up between them, but that she never knew his name. They used to resort to some place in the vicinity of the station. She became pregnant, and was about four months pregnant when she told him of it, and according to her statement he operated to procure abortion, taking her with him into a private room, and using some instrument. The girl went home to Peckham Rye, and there an actual abortion resulted, a fœtus of between four and five months being delivered. That is perhaps the most dangerous stage at which to procure an abortion, because the walls of the uterus are extremely thin, and do not contract well on the bleeding vessels, and so hæmorrhage and septic peritonitis are liable to occur. In this case, septic peritonitis set in, the girl became very ill, and was apparently dying. The police were at once called in, and her dying declaration taken down in the usual form, " I ———, believing that death is about to occur, and that recovery is absolutely impossible, make this statement." She then stated the whole story to the police, and told them further that she was to have met the man that very evening near Victoria Station. The police immediately sent up to Victoria Station, and at the appointed time and place a gentleman appeared. One of the detectives asked him if he was waiting for someone with such and such a name, and he said " Yes." A police inspector stepped forward, and said, " I arrest you for attempting to procure abortion." He at once took to his heels, but after a short chase was captured. He was taken to Peckham Rye in order to be identified. He was taken into her bedroom shortly before the girl actually died. It is not an uncommon thing in a person dying from septicæmia to feel better just prior to the demise. She identified the man and said, " That is the man who procured abortion." Two questions were then put to her. First, " Do you fear death?" She answered, " No." Second, " Do you expect to recover?" She answered, " I do."

These two answers invalidated the dying declaration. It was held that although the previous declaration was a valid one, the subsequent statement invalidated the former declaration, with the result that there was an acquittal. That was the only case, that I know of, in which a dying declaration was invalidated by the subsequent statement of the person dying. This case created a good deal of interest at the time, owing to the fact that a valid dying declaration was subsequently invalidated.

The next case was a starvation case, Reg. *v.* Nichols, 1898. It occupied something like four or five days at the Old Bailey. In it an elderly lady was accused of starving a servant girl. It was a cruel case, and she was found guilty, and sentenced to penal servitude. The points of interest in the case were these. It was an obvious case of starvation, the post-mortem examination showing all the classical signs. The interesting point about the case was that we did not know what the defence would be. The prisoner was extremely well defended by Mr. Marshall Hall, accompanied by a very able junior, and therefore we, on the part of the Crown, knew that some clever defence would be made; but we did not know what it was likely to be. I had therefore to be in court all the time in readiness to meet whatever might be set up. I had already told counsel for the prosecution that in all probability the defence would be either that the girl died from tuberculosis, for a cavity had been found in one of the lungs, or from diabetes. The latter was, in fact, the actual defence. At the post-mortem examination, the bladder was perfectly empty, and there was no chance of examining the urine. That is a point to note in starvation cases. Open the bladder and remove any urine present, so that it may be analysed. In cases of genuine diabetes, there are certain degradation products present, acetone, diacetic ether, and oxybutyric acid. In connection with starvation, some of these degradation products of fat metabolism frequently appear in the urine. The sugar and the acetone and the other bodies which appear in true diabetes are products of a retrograde fatty metabolism. In starvation there is a waste of the fat, with the result that there are certain products of that retrograde metabolism of fat present in the urine, and therefore in starvation it is not uncommon to find sugar and acetone in the urine. I have never found the two other bodies, but these facts very much complicated the answer to the defence. The bladder was empty at the time of death, and therefore everything had to be established by other methods. Dr. Rolleston, of St. George's Hospital, was called for the defence, and he gave his evidence in a most able manner. If anything could have convinced the jury that the patient died of diabetes, it was the fair way and admirable manner in which his evidence was given. The following were the material points which broke down the defence of diabetes. In the first place, in cases of diabetes, the intestinal mucous membrane is generally thickened; in the case of this girl, the intestinal mucous membrane was as thin as tissue paper, and when spread over a newspaper the print could be read through it. That is a sign of starvation. Secondly, in the majority of cases of death from diabetes, the liver is either enlarged or at least not appreciably diminished in weight. In this case the liver was remarkably small, as it is in cases of starvation. Thirdly, in diabetes the kidneys are enlarged. In this case they were small, as they generally are in cases of starvation. Fourthly, in diabetes, the heart is generally normal in size. In this case the heart was small and wasted, as it always is in cases of starvation. It was found necessary to put back into the witness-box the fellow servant of the girl, who had slept in the same room with her for many months before her death, and from her we could obtain no history of polyuria. The defence set up was therefore broken down, although we were not able to show any examination of the urine.

Another case of interest is one which illustrates the question as to what is an abortifacient. I was concerned in a very large number of trials for abortion. Some of these were of extreme interest, and perhaps I shall astonish some of you when I mention the evidence I gave as to what constituted a dangerous abortifacient. The case I refer to is Reg. *v.* Fox and others (Madame Frain), 1899. When this case came before the magistrate he refused to commit the accused for trial; but the Attorney-General, by virtue of the power which he possesses, had them arraigned at the Old Bailey. The Attorney-General who conducted the prosecution is the present Lord Chief Justice. It was an extremely interesting case, both from the legal and from the medico-legal points of view. As you know, at one time advertisements were common in the papers of articles suitable for ladies and warranted to remove obstructions. They were so worded that it was just possible for the vendors to state that the reference was only to obstructions preventing the menstrual flow, without any idea of the question of abortion. This had been going on in a great number of papers for many years, and at last the Public Prosecutor decided to take proceedings. There was no Madame Frain; the company consisted of some men and a woman who carried on business at Hackney Road under that style. They kept a kind of herbal institute, and sold Madame Frain's female mixture and pills. I was supplied with the medicine and the pills. The liquid medicine gave me the most difficult task in connection with analytical work I have ever had. It is easy enough in mixtures to detect mineral substances in them, but not so easy when you are given a coloured medicine of unknown constituents. I was able to prove that it did not contain any known abortifacient. It contained no mineral constituents whatever, except a little vegetable ash. After working at it a long time I came to the conclusion that it was a decoction of some vegetable drug, but I could not tell what it was. It was a dark-coloured mixture, perfectly clear, with no sediment. At last I thought I would centrifugalise some of it, and having done so a very tiny amount of deposit came down, which I microscoped and found to consist of a few starch cells of a kidney shape. I then found out that kidney-shaped starch granules only occur in five known drugs. I made decoctions of the five, and one of them at once answered in its smell and appearance to this medicine. That was the false unicorn root, occasionally used in America. The counsel for the defence knew the composition, and it was a great surprise when I told the court that the mixture contained the false unicorn root, and the oil of pennyroyal. There was no proof whatever that this was an abortifacient, and therefore I unhesitatingly gave evidence that the mixture was perfectly harmless. The conviction was obtained on account of the pills, which contained aloes, jalap, and sulphate of iron. The pills contained three grains of aloes each. I stated that aloes was a dangerous drug to administer to pregnant women, that it was liable to cause abortion, especially in repeated doses, and especially in women liable to abort, and in whom even small doses of aloes may procure abortion. There are many women who have got into the habit of aborting, and who abort on the slightest provocation. With such women six grains of aloes might bring on abortion, and therefore it is quite right to say that aloes is a true abortifacient. It was on the strength of selling these aloes pills that the conviction was obtained.

The next case illustrates the remarkable state of the law as regards patents. A specification was obtained on March 3, 1897, and executed on May 1 of the same year, under the name of Wallace's Specific. Wallace's Specific was to be taken in half-teaspoonful doses, but on the principle that if a small dose does good a large dose does better, an unfortunate individual took the whole bottleful and died. I examined the "specific," and I found that it was a tincture of aconite, made with whisky instead of rectified spirit. The strength of the B.P. tincture of aconite gives 1-25th of a grain of pure aconitine, the most powerful poison known, and this tincture gave 1-27th.

A case in which the careful observation of bloodstains enabled me to reproduce the probable scene of a murder, was that of Reg. *v.* Edwards, 1903, one of the last of my cases before I resigned my Home Office appointment. A certain Edwards killed three people in Camberwell, and, after dismembering the bodies, carried them to Leytonstone and buried them in a back garden. He was arrested when attacking another man. I went with the police to the house in Camberwell, and examined the rooms. It was a very interesting case, as the bloodstains practically enabled me to describe in what way the tragedy had taken place. When an artery has been opened during life, blood spurting on to the wall is thrown in drops, so that the lower part contains most of the blood clot. The stain is a pear shape, the point of the pear being at the top. In two of the rooms, many of the bloodstains which had been projected from the living arteries were found. On the right of the fireplace in the front room underneath the mantelshelf, there were several such bloodstains projected from one or more arteries during life, and they had been projected from left to right. I stated that in all probability one of the victims was attacked from behind whilst sitting on the left side of the fireplace, that the victim's head had fallen downwards and forwards, and so the blood from the severed arteries spurted under the mantelshelf. In all probability the victim recovered, and made a rush to the window in order, no doubt, to call attention, because close to the window I found bloodstains projected on to the wall and the window pane at a level of two and a half feet higher than the mantelshelf, showing that the victim was in an erect position when she was struck down by the final blow, and there she died. Then the murderer turned his attention to the man, who, evidently hearing the fall of the woman on the floor came upstairs to see what was wrong. He was attacked at the top of the stairs, and much projected blood was found there. How was it possible to tell that the man was the last one to be murdered of the three? The murder of the three persons was done by one weapon, a sash weight. At the end of the weight I found three hairs fixed by the coagulated blood. They corresponded with the hair of the man and not with the woman, and therefore the man must have been struck last.

